# Coaxial Electrospray of Curcumin-Loaded Microparticles for Sustained Drug Release

**DOI:** 10.1371/journal.pone.0132609

**Published:** 2015-07-24

**Authors:** Shuai Yuan, Fan Lei, Zhongfa Liu, Qingping Tong, Ting Si, Ronald X. Xu

**Affiliations:** 1 Department of Precision Machinery and Precision Instrumentation, University of Science and Technology of China, Hefei, Anhui, 230027, People’s Republic of China; 2 Department of Modern Mechanics, University of Science and Technology of China, Hefei, Anhui, 230027, People’s Republic of China; 3 College of Pharmacy, The Ohio State University, Columbus, Ohio, 43210, United States of America; 4 Department of Ultrasound, the 105th Hospital of PLA, Hefei, Anhui, 230031, People’s Republic of China; 5 Department of Biomedical Engineering, The Ohio State University, Columbus, Ohio, 43210, United States of America; The University of Tennessee Health Science Center, UNITED STATES

## Abstract

Curcumin exhibits superior anti-inflammatory, antiseptic and analgesic activities without significant side effects. However, clinical dissemination of this natural medicine is limited by its low solubility and poor bio-availability. To overcome this limitation, we propose to encapsulate curcumin in poly(lactic-co-glycolic acid) (PLGA) microparticles (MPs) by an improved coaxial electrospray (CES) process. This process is able to generate a stable cone-jet mode in a wide range of operation parameters in order to produce curcumin-loaded PLGA MPs with a clear core-shell structure and a designated size of several micrometers. In order to optimize the process outcome, the effects of primary operation parameters such as the applied electric voltages and the liquid flow rates are studied systemically. *In vitro* drug release experiments are also carried out for the CES-produced MPs in comparison with those by a single axial electrospray process. Our experimental results show that the CES process can be effectively controlled to encapsulate drugs of low aqueous solubility for high encapsulation efficiency and optimal drug release profiles.

## Introduction

Curcumin (diferuloylmethane, 1,7-bis(4-hydroxy-3-methoxyphenyl)-1,6-heptadiene-3,5- dione) is a yellow pigment derived from the *Curcuma longa* plant [1,2]. It was first isolated in 1815 and its chemical structure was first determined in 1910. Many studies in the past decades have revealed that curcumin exhibits anti-inflammatory, antiseptic and analgesic activities in the treatment of various diseases, such as chronic wounds and cancers [1–4]. However, broader clinical applications of this promising natural drug are limited by its instability in the light, poor oral bio-availability *in vivo* and lack of solubility in aqueous solvents [5,6].

In order to overcome the above limitations and to enhance the bio-availability of curcumin, many formulation techniques and carrier materials have been explored [5–7]. Among them, poly(lactic-co-glycolic acid) (PLGA) microparticles and nanoparticles provide a biodegradable carrier platform for sustained release of curcumin with improved bio-availability [7,8]. Originally approved by Food and Drug Administration (FDA) of the United States for implantation applications, PLGA has been explored as a drug delivery carrier in many oncologic, ophthalmologic, and other medical applications [7–10]. Emulsification is one of the most commonly used processes to fabricate the drug-loaded PLGA MPs [9,10]. Despite its simplicity, this process has several disadvantages, such as the low encapsulation efficiency, the broad particle size distribution, and the process-induced denaturation of the protein cargos.

Electrospray (ES) is the alternative microencapsulation process with the potential to overcome the existing limitations of emulsification [11–13]. The process produces monodisperse particles with size ranging from sub-micrometers to hundreds of micrometers by applying a high positive voltage between a needle and the ground [11,12]. In an ES process, the electric force and the surface tension of the liquid meniscus are balanced to form a conical shape (i.e., “Taylor cone”) at the needle tip. Previous studies have demonstrated the technical feasibility for ES fabrication of multifunctional MPs with improved quality and productivity [11–13]. However, a single axial ES process mixes drugs with carrier materials without forming a protective core-shell structure. Consequently, the produced MPs have suboptimal drug release profiles, typically with an initial burst drug release.

The coaxial electrospray (CES) process has the potential to overcome the above limitations and enable effective encapsulation of proteins, drugs and contrast agents with high efficiency, minimal loss of biological viability, and outstanding control of core-shell architecture [14–16]. The CES process produces double-layered MPs by exposing both the cargo (inner) flow and the carrier (outer) flow from a coaxial needle to an elevated electrical field. At a certain voltage threshold, a Taylor cone forms and the jet of liquids (both inner and the outer flows) is broken into double-layered MPs. The CES technique was first introduced in 2002 when Loscertales *et al*. fabricated the monodisperse capsules in a range of diameter from 0.15 to 10 micrometers by generating stable coaxial jets of two immiscible liquids [15]. Since then, many fundamental experimental and theoretical investigations have been carried out to further enhance the process productivity and quality [17–22]. In 2008, Xie *et al*. developed a CES process to encapsulate protein-based drugs (BSA) in biodegradable polymeric MPs [23]. In 2010, Nie *et al*. fabricated core-shell microspheres by CES for sequential and parallel release of drugs (i.e., paclitaxel and suramin) [24].

This paper reports our recent effort of optimizing the CES process parameters to encapsulate curcumin in PLGA MPs for the high encapsulation rate and the improved drug release profile. The primary operation parameters of the process, such as the applied electric voltages, the inner flow rate, and the outer flow rate, were studied systemically for the stable cone-jet shape and the uniform MP size distribution. The morphology and the core-shell structure of the MPs were confirmed by scanning electron microscopy (SEM) and confocal microscopy. The drug release profile of the produced MPs was evaluated by an *in vitro* experiment and compared with that of MPs fabricated by a single axial electrospray process.

## Materials and Methods

### 1. Materials

Curcumin (C_21_H_20_O_6_) was purchased from Sunny Bio-tech Co. Ltd (Shanghai, China). PLGA (50:50, Mw = 10,000) and PLGA (50:50, Mw = 50,000) were obtained from Shandong Institute of Medical Instrument (Shandong, China). Poly vinyl alcohol (PVA, Mw = 9,000–10,000), Nile-red, coumarin-6, acetone (99.9% high-performance liquid chromatography (HPLC) grade), acetic acid (99.9% HPLC grade) and acetonitrile (99.9% HPLC grade) were purchased from Sigma Aldrich Chemistry (USA). Alcohol and ethyl acetate were purchased from Sino-pharm Chemical Reagent Co. Ltd (Shanghai, China). Deionized water was produced by a pure infinity water purification system (Barnstead International, Dubuque, IA).

### 2. Experimental CES setup

**Fig 1:**
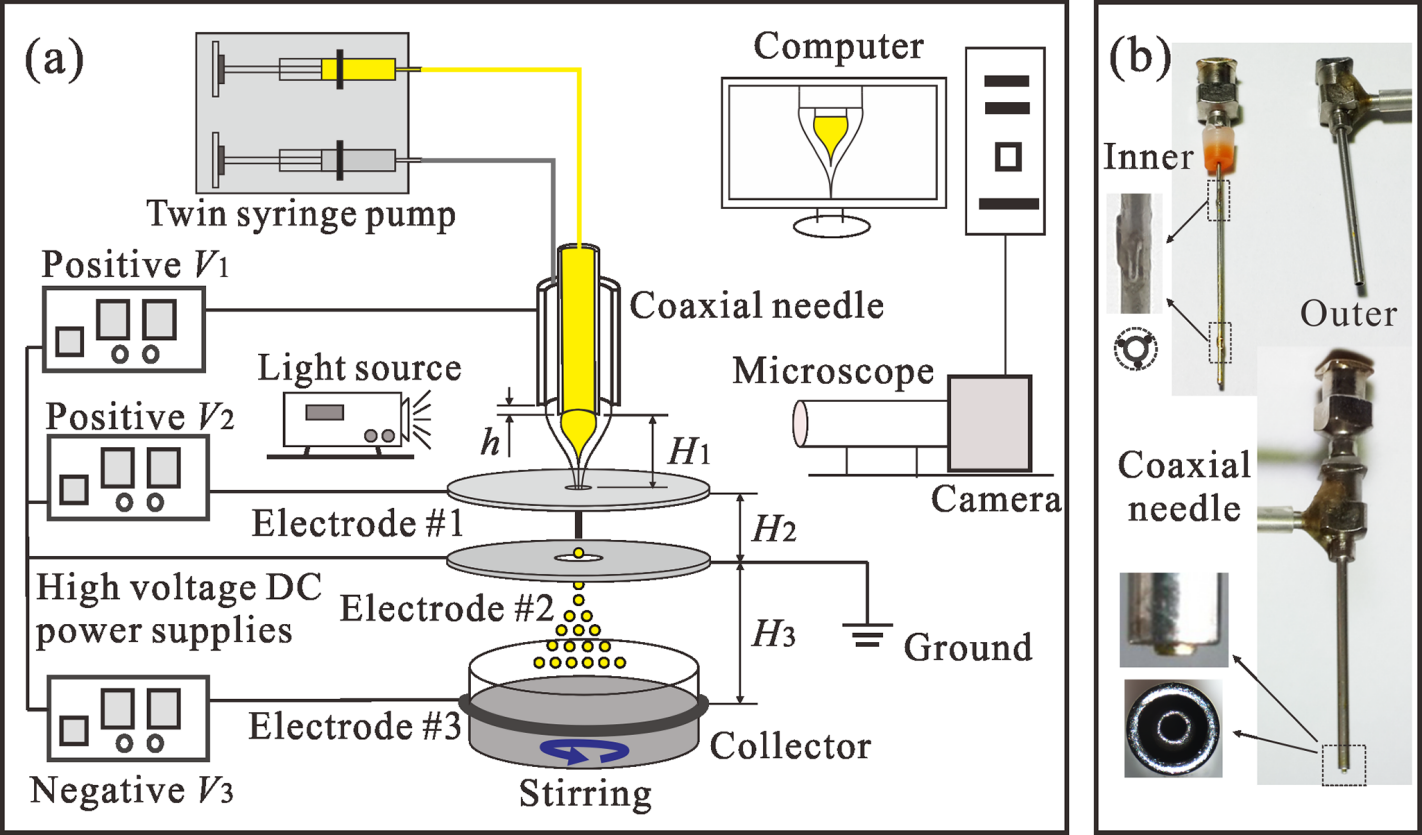
Experimental CES system for fabricating drug-loaded MPs. (a) Schematic of the CES setup including a droplet generation module (twin syringe pump, coaxial needle, electrodes with high voltage power supplies and ground), a droplet collection module (collector with magnetic stir bar), and a process monitoring module (light source, microscopic lens combined with CCD camera and computer); (b) Pictures of the inner needle, the outer needle and the assembly of the coaxial needle.

The experimental CES system consists of a droplet generation module, a droplet collection module, and a process monitoring module, as shown in Fig 1. The droplet generation module includes a coaxial needle, three ring electrodes, a twin syringe pump (Pump33, Harvard Apparatus, MA, USA) and three high voltage DC power supplies (positive and negative voltages, Gamma High Voltage Research, Inc., FL, USA). The coaxial needle (Fig 1b) consists of an inner needle (inner diameter: 0.33 mm; outer diameter: 0.64 mm) and an outer needle (inner diameter: 1.01 mm; outer diameter: 1.48 mm). The tips of the inner and the outer needles are flattened and their edges are rounded. Six short and thin silver wires are welded uniformly on the outside surface of the inner needle by a laser beam welding process (pulsed Nd: YAG laser, 1 Hz, averaged output 300 W). As a result, the positions of the inner and the outer needles (with a vertical distance of *h* between their tips in the range of -0.3~0.5 mm) can be adjusted for high concentricity and the coaxial needle can be readily assembled, aligned and cleaned [25,26]. The electrode #1 is placed underneath the needle assembly with a small vertical distance of *H*_1_ (about 2~4 mm) from the inner needle tip and the electrode #2 is placed underneath the electrode #1 with a vertical distance of *H*_2_ (about 10~50 mm). The ring electrode #3 is fixed around the collector with a vertical distance of *H*_3_ (about 50~150 mm) from the electrode #2. The electrode #1 is used to control the stability of the coaxial cone, and the electrode #3 is used to enhance the movement of the resultant droplets for collection. The electrode #2 is grounded as an auxiliary electrode, which can prevent the droplets flying back to the electrode #1, resulting in a high collection ratio in the process. The droplet collection module includes a beaker containing 80 mL 0.1 w% PVA water solution and a magnetic stir bar driven by a stirring apparatus (Model 78HW-1, China). The process monitoring module includes a 3 kHz strobe flashlight, a charge-coupled device camera (Allied vision technologies, Inc., MA), a computer and a microscopic lens (Model VT-7DS-2CD, Hangzhou, China). The dynamic behavior of the coaxial cone-jet structures can be monitored in real time and stored into data files for processing later.

### 3. Particle preparation and characterization

#### Droplets formation

Under the elevated electric fields, a double-layered Taylor cone can be formed at the tip of the coaxial needle. The coaxial liquid jet is issued from the vertex of the cone, broken into core-shell droplets, and partially dried after solvent evaporation. The flow modes of the CES process are identified and the curcumin-loaded PLGA droplets are fabricated in a stable coaxial cone-jet mode. For the purpose, different groups of the outer and the inner liquids are prepared. Generally, 5~10 w% PLGA (50:50, Mw = 10,000) ethyl acetate solution and 2~5 w% curcumin acetone solution are used. In particular, to improve the viscosity of the inner flow, PLGA (50:50, Mw = 50,000) is dissolved into the inner solution with a concentration of 0.5~2 w%. The operation parameters are varied in a wide range, i.e., the outer liquid flow rate (*Q*_out_): 0.1~5 mL/h, the inner liquid flow rate (*Q*_in_): 0.1~5 mL/h, the applied electric voltages: (*V*_1_) 0~10 kV, (*V*_2_) 0~5 kV (*V*_3_) -10~0 kV. In addition, the geometrical parameters of the CES device including the vertical distances between the inner and the outer needles, the electrodes #1, #2 and #3 (*h*, *H*_1_, *H*_2_, *H*_3_) are adjusted to enhance the stability of the CES process.

#### MP preparation

The curcumin-loaded PLGA MPs are prepared in several steps. After the breakup of the coaxial liquid jet, the droplets are collected in a beaker containing a stirred PVA water solution. Considering that the droplets are still unstable and fragile before the organic solvents volatilize completely, the PVA solution is stirred for 2 hours and then stored at 4°C for 12 hours in order to form a hard PLGA shell. These MPs are centrifuged by a TDZ4A-WS Centrifuge (Hefei, China) at 4,000 rpm for 5 minutes. After the supernatant is discarded, the sediment is washed by the deionized water for three times, frozen to -80°C in a Zhongke Meiling ultra-low temperature freezer (Hefei, China) for 3 hours, and lyophilized in a LGJ-10 freeze dry system (Beijing, China) for 24 hours. Dried MPs are stored in a light resistant container at 4°C for further use.

#### MP morphology

An SIRION 200 Scanning electron microscopy (SEM) system (FEI, Holland) is used to observe the morphologic features of the produced curcumin-loaded PLGA MPs. The SEM is operated at +10 kV in high vacuum mode. Before SEM imaging, the MPs are gently placed on aluminum tubs and gold coated for 180 seconds. The core-shell structure of the produced MPs is verified by a LSM710 Confocal laser scanning microscopy (CLSM) system (Zeiss, Peabody, MA). Nile red (0.01 w%) is added into the outer PLGA liquid and coumarin-6 (0.01 w%) into the inner curcumin solution for imaging. The MPs are observed by CLSM 63× objective with a 9.7× zoom. The excitation wavelengths for Nile red is 549 nm and for coumarin-6 is 450 nm. The emission is acquired between 400~500 nm and 600~700 nm.

### 4. Loading rate and encapsulation efficiency

A method that couples high performance liquid chromatography (HPLC) and ultraviolet (UV) detection is used to determine the curcumin concentration in the produced MPs with standard protocols [27]. An isocratic elution with a solvent mixture of 0.12% acetic acid, 59.88% deionized water and 40% acetonitrile is used for HPLC-UV measurement. For HPLC testing, a Waters symmetry C18 column (4.6 mm × 150 mm × 5 μm) is connected to the HPLC system and the mobile phase consists of a 0.2% acetic acid aqueous and an acetonitrile solution. The liquid flow rate of the acetic acid aqueous and the acetonitrile is 1.2 mL/min and 0.8 mL/min, respectively. The wavelength of detection is 422 nm. 50 mg solidified PLGA MPs are dissolved into 5 mL acetone solution after stirring for 12 hours. Loading rate (LR) (%) and encapsulation efficiency (EE) (%) are derived by the following equations:

LR= Mass of measured curcumin / Total mass ofPLGA−curcumin×100%,

EE = Mass of measured curcumin / Totalmass ofcurcuminin PLGA−curcumin×100%.

### *5*. *In vitro* drug release

*In vitro* drug release test is carried out for curcumin-loaded PLGA MPs produced by the CES process and compared with those produced by the single axial electrospray (ES) process. Each sample of 40 mg dried MPs is dispersed in 1 mL deionized water and sealed in a dialysis bag for drug release testing. The dialysis bags are placed in the vials filled with 200 mL 50:50 (v/v) alcohol aqueous solution. The vials are placed in a constant temperature shaker at 37°C and shaken at 50 rpm. To prevent light-induced degradation and water evaporation, the vials are covered by aluminum foil. At the scheduled time interval of 0, 0.5, 1, 2, 4, 6, 9, 12, 24 hours, and 1, 2, 4, 8, 16, 24, 32, 40 days, 1 mL of each sample is taken for HPLC-UV measurement and compensated by 1 mL fresh release medium.

## Results and Discussion

### 1. Flow modes in CES process

**Fig 2:**
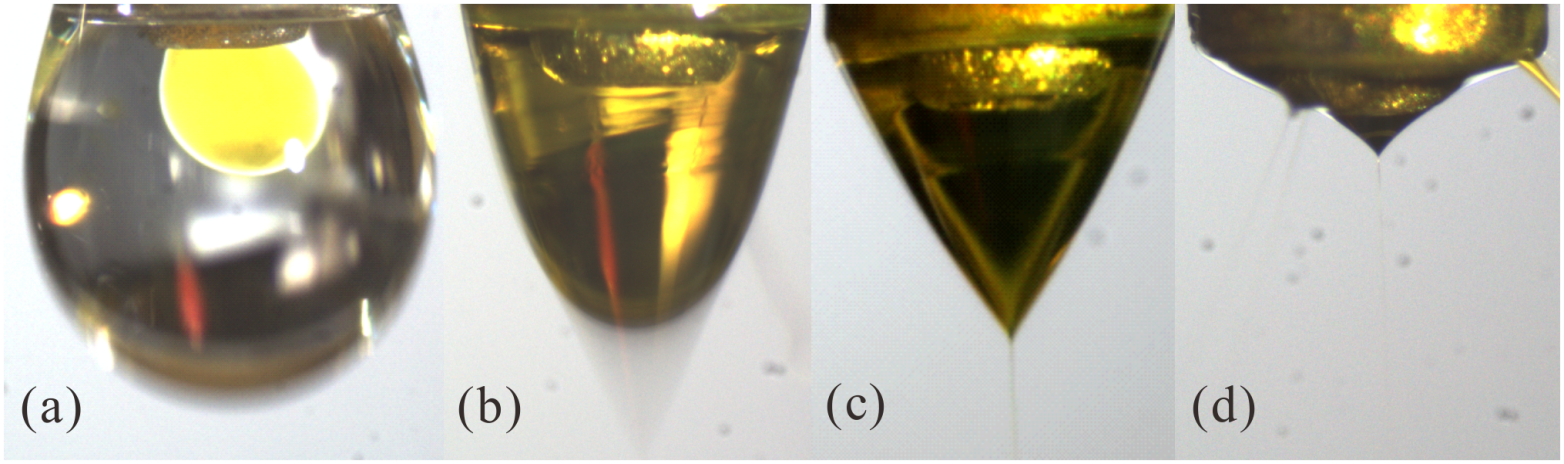
Four sequential flow modes of CES with the increased positive voltage. (a) Dripping mode, *V*_1_ = 1.5 kV; (b) Coning mode, *V*_1_ = 3.5 kV; (c) Stable cone-jet mode, *V*_1_ = 5 kV; (d) Multi-jet mode, *V*_1_ = 7 kV. The applied voltages: *V*_2_ = 1.5 kV, *V*_3_ = -5 kV; The outer liquid: 10.0 w% PLGA (Mw = 10,000) in ethyl acetate solution, *Q*_out_ = 1.0 mL/h; The inner liquid: 4.0 w% curcumin and 1.0 w% PLGA (Mw = 50,000) in acetone solution, *Q*_in_ = 1.0 mL/h; The vertical distances: *h* = 0.1 mm, *H*_1_ = 2.5 mm, *H*_2_ = 20 mm, *H*_3_ = 100 mm.

We have previously studied the different flow modes of a CES process affected by the process parameters, such as the applied electrical voltages [19]. In this work, the flow modes of the process are identified by changing the positive voltage *V*_1_ while keeping the other parameters constant. Fig 2 shows the acquired images of the following four flow modes at different voltage levels: dripping mode, coning mode, stable cone-jet mode, and multi-jet mode. The inner and the outer liquid flow rates are equally set to be 1.0 mL/h. A positive voltage of *V*_2_ = 1.5 kV and a negative voltage of *V*_3_ = -5 kV are applied on the electrode #1 and the bottom ring electrode #3, respectively. When the value of *V*_1_ is smaller than 2 kV, the dripping mode can be observed, in which the electric force is weak so that the inner and the outer liquids maintain a nearly spherical shape because of the surface/interface tension (Fig 2a). As *V*_1_ increases from 2 kV to 4 kV, the cone becomes shaking with a coning shape, which is referred to as the coning mode (Fig 2b). The stable cone-jet mode occurs when *V*_1_ increases from about 4 kV to 6 kV (Fig 2c). In this mode, the coaxial cone can be always stable, resulting in MPs with good morphology. As the applied positive voltage further increases, the cone becomes small and the liquid jet turns to multiple branches (Fig 2d). This mode is named as the multi-jet mode. It should be mentioned that when the applied positive voltage *V*_2_ and the liquid flow rates *Q*_out_ and *Q*_in_ vary, the critical values of *V*_1_ for different modes will be different. Besides, the applied negative voltage *V*_3_ hardly changes the transition between these flow modes, and it mainly affects the collection of the resultant droplets. In this work, we mainly produce curcumin-loaded PLGA MPs under the stable cone-jet mode, which has been identified as the mostly suitable mode for microencapsulation in previous studies [23,24].

### 2. Characteristics of stable cone-jet mode

The CES process is challenged by its complex physics with multiple design, materials and operation parameters contributing to the outcome. As these parameters change, the shape of the double-layered structure menisci will vary in order to maintain the balance between the electric force, the surface/interface tension and the inertia force at the flow interfaces. We have previously performed the experimental and the theoretical studies to optimize the CES process for the fabrication of PLGA MPs [18]. We have also reviewed the recent advances in design, modeling and control of the CES process for microencapsulation [19]. However, the CES process parameters for the specific MP materials have not been optimized yet for stable coaxial cone-jet mode and successful droplet formation. In this work, we study the effects of the primary operation parameters on the coaxial cone-jet structure and the droplet diameter. The drug-loaded PLGA MPs produced at the optimized process parameters are then chosen for the drug release testing.

**Fig 3:**
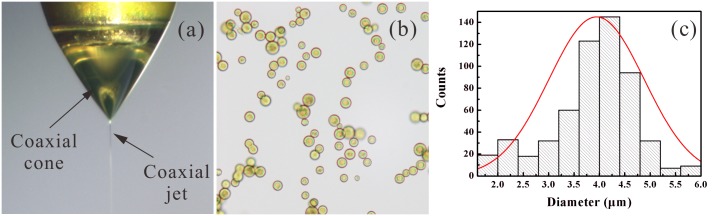
The stable cone-jet mode and the curcumin-loaded PLGA droplets produced by a CES process. (a) The morphology of the coaxial cone and the coaxial jet; (b) Microscopic image of curcumin-loaded PLGA droplets directly collected by a glass slide, right after the breakup of the coaxial jet; (c) The size distribution of the collected droplets. The applied voltages: *V*_1_ = 4.8 kV, *V*_2_ = 1.5 kV, *V*_3_ = -8 kV; The outer liquid: 10.0 w% PLGA (Mw = 10,000) in ethyl acetate solution, *Q*_out_ = 1.0 mL/h; The inner liquid: 4.0 w% curcumin and 1.0 w% PLGA (Mw = 50,000) in acetone solution, *Q*_in_ = 1.0 mL/h; The vertical distances: *h* = 0.2 mm, *H*_1_ = 2.5 mm, *H*_2_ = 20 mm, *H*_3_ = 80 mm.

Fig 3 presents a typical image of the stable coaxial cone-jet structure and a microscopic image of resultant droplets directly collected after the breakup of the coaxial jet. In the stable cone-jet mode, two parallel flows can be clearly observed in the coaxial cone, where the inner flow is curcumin solution (Fig 3a). It is very important to keep this stable double-layered flow structure in the CES process in order to encapsulate the inner curcumin cargo in the outer PLGA shell completely, as shown in Fig 3(b), indicating a nearly 100% encapsulation efficiency in theory. The yellow core is curcumin in acetone and the shell is PLGA solution where part of solvent has evaporated. The mean diameter of these droplets is 3.94 μm with a standard deviation of 0.51 μm (Fig 3c). The inner and the outer liquids are 10.0 w% PLGA (Mw = 10,000) ethyl acetate solution and 4.0 w% curcumin and 1.0 w% PLGA (Mw = 50,000) acetone solution. It is worth noting that two kinds of PLGA with different molecular weights (i.e., Mw = 10,000 and 50,000) are used to prepare the inner and the outer solutions, and PLGA (Mw = 50,000) is dissolved into the inner solution. On one hand, the viscosity of the inner solution can be increased to minimize the mixing between two parallel organic solutions in the droplet formation process. On the other hand, the curcumin and PLGA are dissolved in the core and are encapsulated together in the shell. Therefore, PLGA with high molecular weight will load the curcumin inside the shell and enhance the stability of the double-layered structure of MPs especially in the post-processing later.

**Fig 4:**
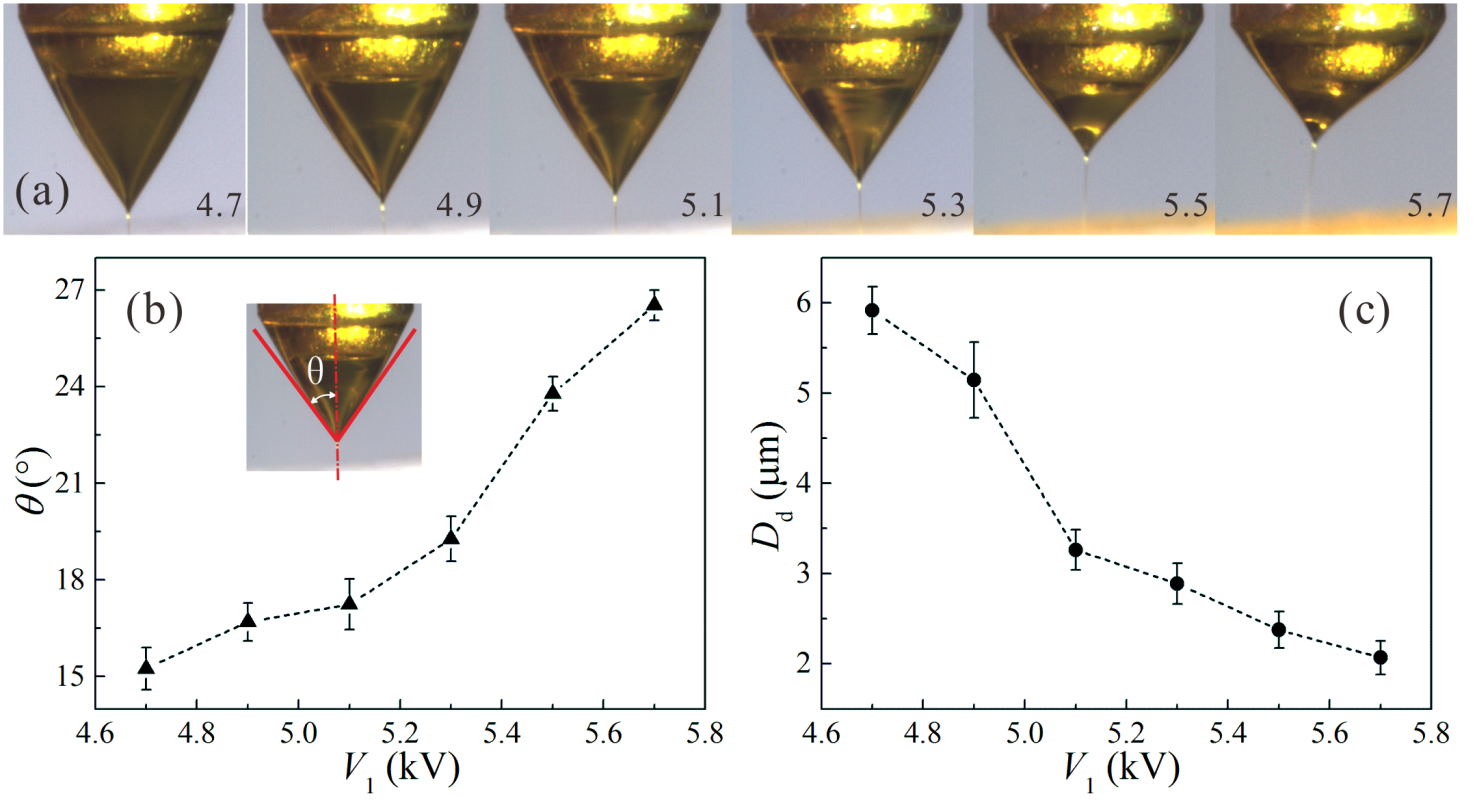
The influence of applied positive voltage *V*_1_ on the stable coaxial cone-jet mode and the size of resultant droplets. (a) Sequence of experimental images showing the stable cone-jet structure changing with *V*_1_; (b) The angle of the cone *θ* as a function of *V*_1_; (c) The diameter of the resultant droplets *D*_d_ as a function of *V*_1_. The applied voltages: *V*_2_ = 1.5 kV, *V*_3_ = -8 kV; The outer liquid: 10.0 w% PLGA (Mw = 10,000) in ethyl acetate solution, *Q*_out_ = 1.0 mL/h; The inner liquid: 4.0 w% curcumin and 1.0 w% PLGA (Mw = 50,000) in acetone solution, *Q*_in_ = 1.0 mL/h; The vertical distances: *h* = 0.3 mm, *H*_1_ = 2 mm, *H*_2_ = 20 mm, *H*_3_ = 80 mm.

As mentioned above, the positive voltage (*V*_1_) applied on the coaxial needle in a CES process plays an important role in the formation of drug-loaded PLGA droplets. Fig 4(a) shows the sequence of experimental images of the stable cone-jet mode changing with *V*_1_. For a group of fixed operation parameters, the outer meniscus is able to maintain a stable conical shape due to the balance between the surface tension, the pressure difference across the liquid-gas interface, and the electric stresses caused by the applied electric field. The bulk free charges are mainly located at the outer interface [14]. As the value of *V*_1_ increases, the coaxial Taylor cone becomes thin and short due to the enhanced electric stresses on the outer interface. Defining a cone angle *θ* as the half angle of the outer meniscus tip, one can measure the value of *θ* as a function of *V*_1_, as plotted in Fig 4(b). The relationship between the diameter of collected droplets and the applied positive voltage can also be obtained, as shown in Fig 4(c). It is indicated that, as the value of *V*_1_ increases from 4.7 kV to 5.7 kV in the stable cone-jet mode at the same feeding outer and inner flow rates of 1.0 mL/h, the cone angle has a growth from about 15° to 27.5°, and the diameter of core-shell structure droplets decreases from about 5.92 μm to 2.07 μm. Because the droplets are collected after the break up of electrified coaxial liquid jets, the droplet size is closely related to the jet diameter and the jet instability [16,18,19]. Qualitatively, as the applied voltage increases, both the normal and the tangential components of electric stresses are enhanced. Therefore, the diameter of the coaxial liquid jet is reduced dramatically and the break up of the coaxial liquid jet is promoted, resulting in the reduction of the collected droplets.

**Fig 5:**
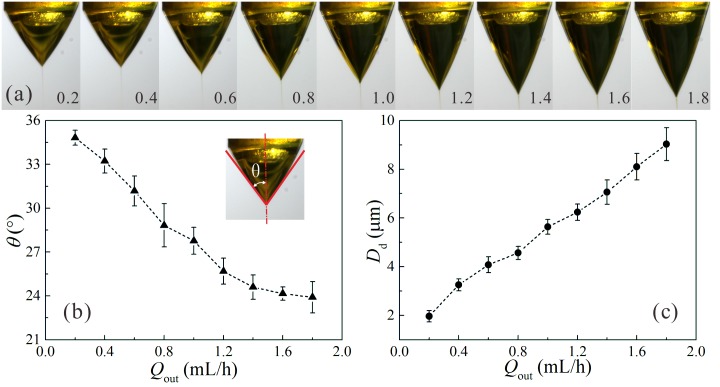
The influence of outer liquid flow rate *Q*_out_ on the stable cone-jet mode and the size of resultant droplets. (a) Sequence of experimental images showing the stable cone-jet structure changing with *Q*_out_; (b) The angle of the cone *θ* as a function of *Q*_out_; (c) The diameter of the resultant droplets *D*_d_ as a function of *Q*_out_. The applied voltages: *V*_1_ = 4 kV, *V*_2_ = 1.5 kV, *V*_3_ = -5 kV; The outer liquid: 10.0 w% PLGA (Mw = 10,000) in ethyl acetate solution; The inner liquid: 4.0 w% curcumin and 1.0 w% PLGA (Mw = 50,000) in acetone solution, *Q*_in_ = 0.2 mL/h; The vertical distances: *h* = 0.3 mm, *H*_1_ = 2 mm, *H*_2_ = 20 mm, *H*_3_ = 80 mm.

Fig 5 presents the influence of the outer liquid flow rate *Q*_out_ on the stable coaxial cone-jet structure and the size of droplets collected just after the breakup of the coaxial liquid jet. The increase of the outer liquid flow rate from 0.2 mL/h to 1.8 mL/h shows a clear tendency with an increase in the diameter of droplets (from about 1.96 μm to 9.03 μm) and a decrease in the cone angle *θ* (from about 35° to 24°), and the coaxial cone is elongated. As the diameter of the coaxial jet is positively correlated with the liquid flow rate [18,19], it is easily understood that the mean diameter of the droplets increases gradually as the value of *Q*_out_ increases. In particular, the droplet diameter increases linearly with the outer liquid flow rate, which is in accordance with the analytical scaling law predicted in previous studies [14]. It was also pointed out that due to the higher viscosity of the inner liquid, the size of the resultant droplets was mainly governed by the break up of the inner liquid jet, and the viscous stresses acting at the liquid-liquid interface was considerable [14]. As a result, the balance of accelerating and drag forces leads to the dependence of the droplet size on the liquid flow rate. The results indicate that the CES process is an effective approach to obtain drug-loaded PLGA droplets with tunable size by simply controlling the liquid flow rates and the applied voltages in the stable cone-jet mode for a given group of materials and a fixed CES device.

### 3. Characteristics of curcumin-loaded PLGA MPs

The curcumin-loaded PLGA droplets collected just after the breakup of the coaxial liquid jet are still unstable and fragile because the organic solvents are not volatilized completely. As the matter of fact, massive fabrication of drug-loaded PLGA MPs in a CES process places a big challenge, especially in the process where the shell is a volatile material. Collecting MPs directly by a hard plate such as aluminum foil will affect the particle morphology and easily form particle aggregation since the collected droplets are not solidified. Collecting MPs using stationary liquids like aqueous solution may introduce surface tension issues that prevent the formation of the spherical MPs going into the collector. To overcome this challenge, the droplets are collected in a stirred 0.1 w% PVA solution to create the “soft landing” environment and effectively avoid the particle aggregation. After several steps including centrifuging, washing and lyophilizing processes, the curcumin-loaded PLGA MPs are solidified with removal of all organic solvents for further use.

**Fig 6:**
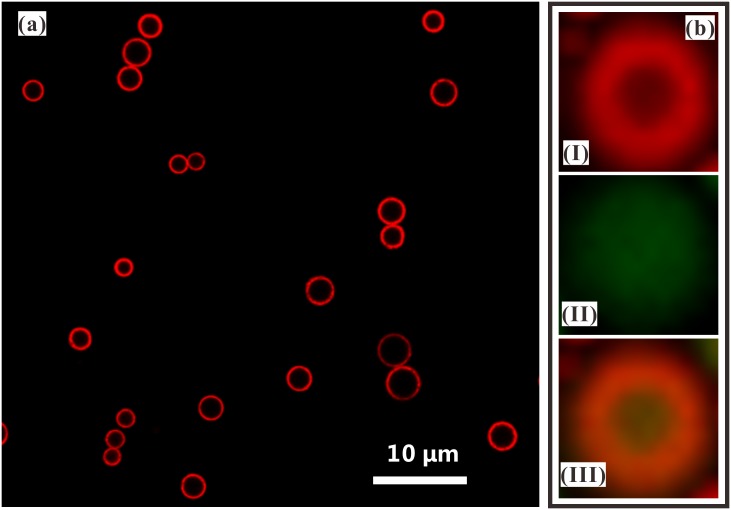
Core-shell structure of curcumin-loaded PLGA droplets produced by CES. (a) Confocal fluorescence microscopic image of droplets showing the shell shape (Nile red 0.01 w% in outer liquid); (b) Confocal fluorescence microscopic image of MPs showing the core (I), the shell (II) and the core-shell shapes (III) (Nile red 0.01 w% in outer liquid and coumarin-6 0.01 w% in inner liquid). The applied voltages: *V*_1_ = 4.8 kV, *V*_2_ = 1.5 kV, *V*_3_ = -5 kV; The outer liquid: 10.0 w% PLGA (Mw = 10,000) in ethyl acetate solution, *Q*_out_ = 1.0 mL/h; The inner liquid: 4.0 w% curcumin and 1.0 w% PLGA (Mw = 50,000) in acetone solution, *Q*_in_ = 0.5 mL/h; The vertical distances: *h* = 0.2 mm, *H*_1_ = 2 mm, *H*_2_ = 20 mm, *H*_3_ = 80 mm.

Confocal laser scanning microscopy (CLSM) and scanning electron microscopy (SEM) have been commonly used to show the core-shell structure of the multilayered MPs. In this work, the morphology of curcumin-loaded PLGA droplets collected in the PVA solution are first observed by CLSM imaging. When the outer PLGA solution is added by Nile red (0.01 w%), a circular shape of the shell can be clearly observed by the CLSM imaging, as shown in Fig 6(a). When the inner curcumin solution is further stained by coumarin-6 (0.01 w%), the core-shell structure with PLGA as the shell and curcumin solution as the core can be imaged by different channels of CLSM system, as indicated in Fig 6(b). The confocal fluorescence images show that the curcumin-loaded PLGA droplets have a satisfactory morphology and a core-shell structure.

**Fig 7:**
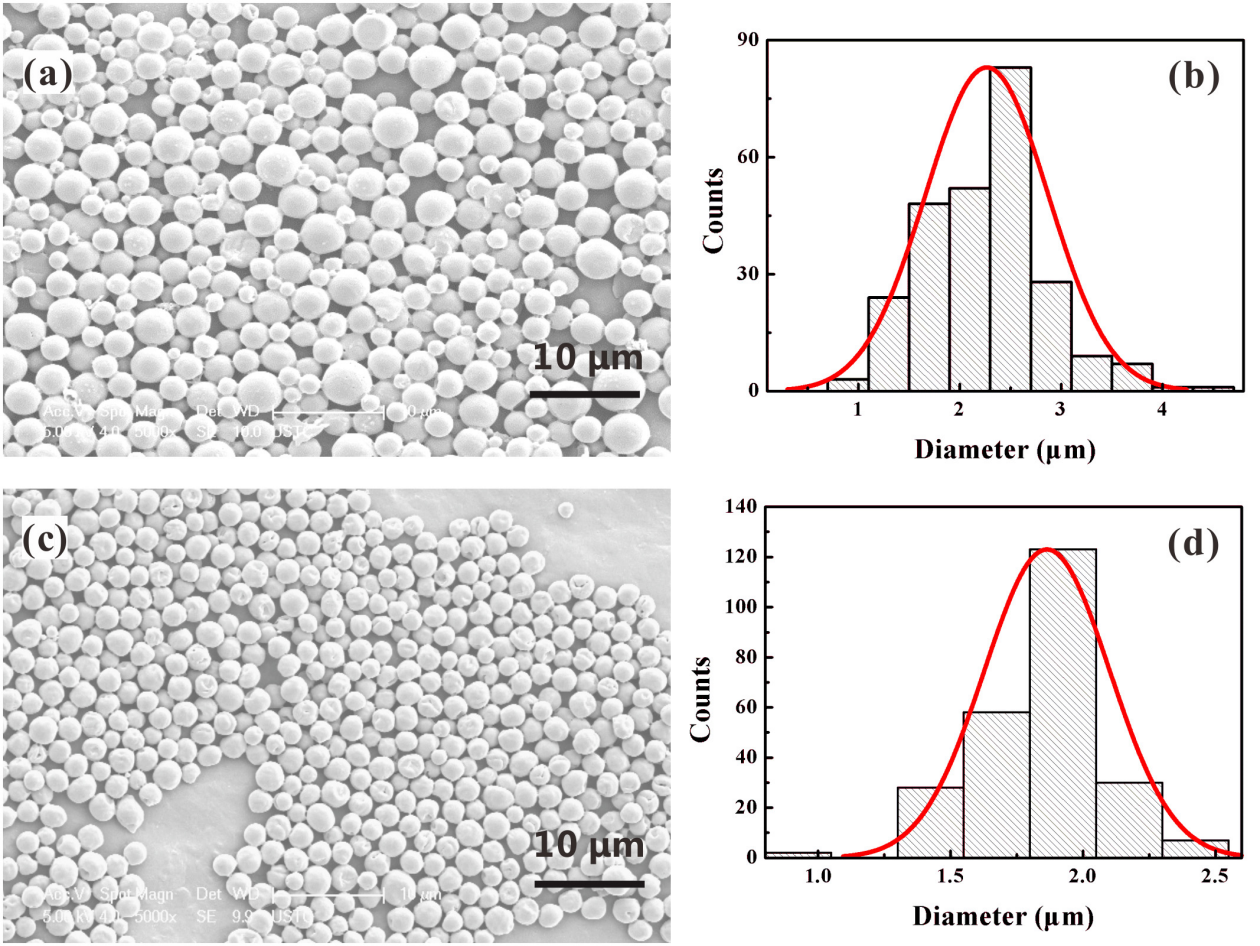
Mass production of curcumin-loaded PLGA MPs produced by CES. (a) SEM imaging and (b) size distribution of the MPs for *Q*_out_ = *Q*_in_ = 1.0 mL/h; (c) SEM imaging and (d) size distribution of the MPs for *Q*_out_ = 1.0 mL/h and *Q*_in_ = 0.5 mL/h. The applied voltages: *V*_1_ = 4.8 kV, *V*_2_ = 1.5 kV, *V*_3_ = -8 kV; The outer liquid: 10.0 w% PLGA (Mw = 10,000) in ethyl acetate solution; The inner liquid: 4.0 w% curcumin and 1.0 w% PLGA (Mw = 50,000) in acetone solution; The vertical distances: *h* = 0.2 mm, *H*_1_ = 2.5 mm, *H*_2_ = 20 mm, *H*_3_ = 80 mm.

Guided by the above analysis, we have fabricated curcumin-loaded PLGA MPs with size ranging from hundreds of submicron to tens of micron by the CES process. To further reduce the size of MPs, a practical method is to increase the applied voltages (especially *V*_1_) and reduce the inner or outer liquid flow rates by maintaining a stable cone-jet mode. As the levels of applied voltages and liquid flow rates vary, the geometrical structures of the CES device (e.g., the vertical distances *h* and *H*_1_) have to be adjusted in order to enhance the stability of the coaxial cone. As a demonstration, Fig 7 presents the SEM imaging of curcumin-loaded PLGA MPs fabricated under different inner liquid flow rates. It can be easily seen that the improved CES process yields MPs with monodisperse size distribution and satisfactory morphology. For an outer liquid flow rate of 1.0 mL/h, as the inner liquid flow rate decreases from 1.0 mL/h to 0.5 mL/h, the mean diameter of MPs varies from 2.25 μm to 1.87 μm. Besides, comparison between Fig 7(a) and Fig 3 shows that the size of these solidified MPs (i.e., the mean diameter is 2.25 μm) is much smaller than that of the droplets directly collected after the breakup of coaxial liquid jet (i.e., the mean diameter is 3.94 μm), although the operation parameters in both figures are the same. The reason definitely lies in the fact that the organic solvents have been completely volatilized in these MPs. As a result, the CES process is available to prepare MPs loading different drugs inside the shell with small size, nearly spherical shape and narrow size distribution, which have great potentials in biomedical and clinical applications.

By optimizing the operation parameters in CES, it is possible to establish a stable coaxial cone-jet mode for both the inner and the outer flows. Since the inner liquid can be completely encapsulated in the outer liquid, the CES process is able to achieve an encapsulation efficiency (EE) theoretically as high as 100%. Possible reasons for the reduction of the actual EE include the stray droplets uncollected in the process and the loss of the drugs during post-processing. In this work, we have carefully prepared 5 samples of curcumin-loaded PLGA MPs fabricated under different operation parameters in CES and measured the EE of drugs using the HPLC-UV method. Meanwhile, the drug loading rate (LR) of these samples are also measured. In the HPLC-UV measurements, each sample is divided equally into 3 tests. The averaged results for these 5 samples are given in Table 1. For comparison, the inner and the outer flow rates (*Q*_in_ and *Q*_out_), the PLGA (Mw = 10,000) concentration in the inner solution and the PLGA (Mw = 50,000) concentration in the outer solution are adjusted separately by keeping the other operation parameters constant in these 5 samples.

**Table 1:** The EE and LR of drugs for curcumin-loaded PLGA MPs fabricated in CES. The applied voltages: *V*_1_ = 4.8 kV, *V*_2_ = 1.5 kV, *V*_3_ = -8 kV; The outer liquid: PLGA (Mw = 10,000) in ethyl acetate solution; The inner liquid: 4.0 w% curcumin and PLGA (Mw = 50,000) in acetone solution; The vertical distances: *h* = 0.2 mm, *H*_1_ = 2.5 mm, *H*_2_ = 20 mm, *H*_3_ = 80 mm.

Sample	*Q*_out_ (mL/h)	*Q*_in_ (mL/h)	Outer PLGA (w%)	Inner PLGA (w%)	EE (%)	LR (%)
**1**	1.0	1.0	10.0	1.0	95.42±1.83	31.80±0.61
**2**	0.8	1.0	10.0	1.0	90.86±1.31	34.95±0.51
**3**	1.0	1.2	10.0	1.0	80.48±0.22	35.53±0.07
**4**	1.0	1.0	6.0	1.0	86.65±1.16	48.78±0.94
**5**	1.0	1.0	10.0	0	89.90±0.31	38.55±0.13

As shown in Table 1, by reducing the supplied flow rate of the outer PLGA solution or increasing that of the inner curcumin solution in a specific range, the measured drug LR becomes large, but the EE level decreases slightly. It can be seen that increasing the concentration of PLGA in the outer or the inner liquids can efficiently increase the EE of curcumin, but will result in a reduction of the drug LR. On the one hand, the measured EE of curcumin for each sample is higher than 80%, depending on the whole fabrication process and the post-processing. The addition of PLGA by increasing the outer PLGA solution flow rate, the outer and the inner PLGA concentrations indeed improves the drug EE, which implies that the drug can be efficiently protected in the core-shell structure MPs by increasing the thickness of the PLGA shell or forming a PLGA matrix inside the shell for inner drug retention. On the other hand, the measured LR of curcumin for each sample is higher than 30%, and one sample among them can reach as high as 48.78%. As the protocols for the MPs fabrication process, the post-processing and the HPLC-UV measurement are nearly the same for these 5 samples and the EE of curcumin for each sample is high, it is easily understood that the drug LR is mainly determined by the weight ratio of curcumin in the total MPs. The results indicate that CES is a more efficient and reliable process for producing drug-loaded MPs with satisfactory morphology, narrow size distribution, high EE and LR of drugs, in comparison with traditional microencapsulation methods such as emulsification.

### 4. Drug release *in vitro* study

**Fig 8:**
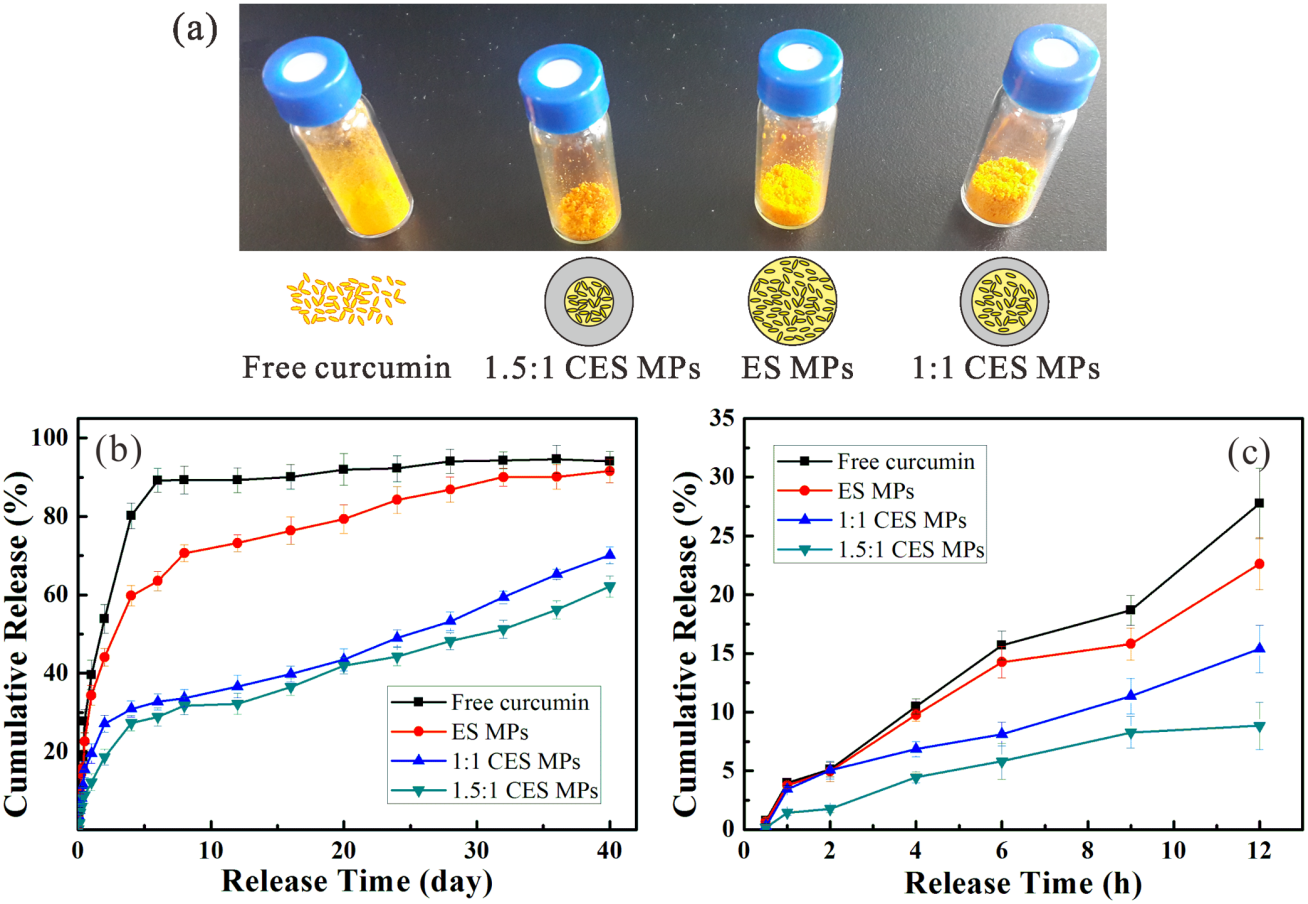
Comparison of the curcumin release profiles versus time at different conditions. (a) Picture of four samples and schematics of corresponding structures of free curcumin, ES and CES MPs; (b) Drug release profile in 40 days of free curcumin and MPs prepared by ES and CES; (c) The enlarged figure of the release profile in first 12 hours.

The curcumin-loaded PLGA MPs are fabricated by the CES process. The drug release profiles of these MPs are tested and compared with those of free curcumin and curcumin-PLGA matrix produced by single axial electrospray (ES), as shown in Fig 8. For two CES-produced MP formulations (as denoted by “1:1 CES MPs” and “1.5:1 CES MPs” in Fig 8), the inner liquid flow rate is the same (i.e., *Q*_in_ = 1.0 mL/h) and the outer liquid flow rate is different (i.e., *Q*_out_ = 1.0 mL/h and 1.5 mL/h, respectively). The outer liquid is 10.0 w% PLGA (Mw = 10,000) in ethyl acetate solution, and the inner liquid is 4.0 w% curcumin and 1.0 w% PLGA (Mw = 50,000) in acetone solution. The applied voltages are *V*_1_ = 4.8 kV, *V*_2_ = 1.5 kV, *V*_3_ = -8 kV and the vertical distances are *h* = 0.2 mm, *H*_1_ = 2.5 mm, *H*_2_ = 20 mm, *H*_3_ = 80 mm, respectively. The ES MPs are fabricated in an ES device, which is similar to the CES one but utilizes a single needle with an inner diameter of 1.01 mm and an outer diameter of 1.48 mm instead of the coaxial needle. The ES liquid is prepared by mixing 1:1 v/v the inner curcumin and the outer PLGA solutions in the CES case. The liquid flow rate is set as 2.0 mL/h and the applied positive voltages are adjusted as *V*_1_ = 5.5 kV, *V*_2_ = 1.5 kV, *V*_3_ = -8 kV to achieve a stable cone-jet mode and a mean droplet diameter of about 4 μm. The fabrication process and the post-processing for obtaining the ES MPs are similar to those in the CES experiments.

The curcumin release profiles of 4 samples measured for 40 days are shown in Fig 8(b). In the first 12 hours, as indicated in Fig 8(c), the drug release of free curcumin and the ES MPs is faster than the CES MPs. As time proceeds, an initial burst drug release and a following slower release can be found for these 4 samples. For the free curcumin, the drug release mainly takes place in about 5 days, involving a significant burst release of about 45% in total in the first day. For the MPs fabricated by a single axial ES process, drug release takes place for about 30 days, and about 60% release occurs in the first 5 days. However, for the CES MPs with the same size as ES MPs, the drug release profile is different, which is also dependent on the morphology of the core-shell structured MPs. For the 1:1 CES MPs, about 30% curcumin release occurs in the first 5 days, and a sustained drug release corresponding to a total of about 70% is found for 40 days. It is easily seen that the 1.5:1 CES MPs release more slowly over the entire period of study because of the increased thickness of the PLGA shell, which indicates that a controlled drug release can be achieved from the CES MPs.

## Conclusion

In this work, curcumin-loaded PLGA microparticles (MPs) are fabricated by an improved coaxial electrospray (CES) process. Four sequential cone-jet modes are observed as the applied voltage increases. Core-shell structured MPs with designated morphologic characteristics and high drug encapsulation efficiency are obtained in the stable cone-jet mode. The core-shell structure of these MPs is clearly visible in both CLSM and SEM images. The size of the MPs can be effectively controlled by tuning the primary process parameters such as the applied electric voltages and the liquid flow rates. The drug release performance of the CES-produced MPs is compared with that of the ES-produced MPs in a 40-day drug release study. The test results show that the CES process yields MPs with improved drug release profiles in comparison with traditional microencapsulation methods. Our study has demonstrated the technical potential of using the CES process to encapsulate therapeutic cargos of poor water solubility for sustained drug release applications.
